# Label noise in subtype discrimination of class C G protein-coupled receptors: A systematic approach to the analysis of classification errors

**DOI:** 10.1186/s12859-015-0731-9

**Published:** 2015-09-29

**Authors:** Caroline König, Martha I Cárdenas, Jesús Giraldo, René Alquézar, Alfredo Vellido

**Affiliations:** 1 Dept. of Computer Science, Univ. Politècnica de Catalunya, C. Jordi Girona, 1-3, Barcelona, 08034 Spain; 2 Institut de Neurociències, Unitat de Bioestadística, Univ. Autònoma de Barcelona, Cerdanyola del Vallès, Barcelona, 08193 Spain; 3 Institut de Robòtica i Informàtica Industrial, CSIC-UPC, Barcelona, 08034 Spain; 4 Centro de Investigación Biomédica en Red en Bioingeniería, Biomateriales y Nanomedicina (CIBER-BBN), Cerdanyola del Vallès, Barcelona, 08193 Spain

**Keywords:** G Protein-coupled receptors, Label noise, Support vector machines, Phylogenetic trees

## Abstract

**Background:**

The characterization of proteins in families and subfamilies, at different levels, entails the definition and use of class labels. When the adscription of a protein to a family is uncertain, or even wrong, this becomes an instance of what has come to be known as a *label noise* problem. Label noise has a potentially negative effect on any quantitative analysis of proteins that depends on label information. This study investigates class C of G protein-coupled receptors, which are cell membrane proteins of relevance both to biology in general and pharmacology in particular. Their supervised classification into different known subtypes, based on primary sequence data, is hampered by label noise. The latter may stem from a combination of expert knowledge limitations and the lack of a clear correspondence between labels that mostly reflect GPCR functionality and the different representations of the protein primary sequences.

**Results:**

In this study, we describe a systematic approach, using Support Vector Machine classifiers, to the analysis of G protein-coupled receptor misclassifications. As a proof of concept, this approach is used to assist the discovery of labeling quality problems in a curated, publicly accessible database of this type of proteins. We also investigate the extent to which physico-chemical transformations of the protein sequences reflect G protein-coupled receptor subtype labeling. The candidate mislabeled cases detected with this approach are externally validated with phylogenetic trees and against further trusted sources such as the *National Center for Biotechnology Information*, *Universal Protein Resource*, *European Bioinformatics Institute* and *Ensembl Genome Browser* information repositories.

**Conclusions:**

In quantitative classification problems, class labels are often by default assumed to be correct. Label noise, though, is bound to be a pervasive problem in bioinformatics, where labels may be obtained indirectly through complex, many-step similarity modelling processes. In the case of G protein-coupled receptors, methods capable of singling out and characterizing those sequences with consistent misclassification behaviour are required to minimize this problem. A systematic, Support Vector Machine-based method has been proposed in this study for such purpose. The proposed method enables a filtering approach to the label noise problem and might become a support tool for database curators in proteomics.

**Electronic supplementary material:**

The online version of this article (doi:10.1186/s12859-015-0731-9) contains supplementary material, which is available to authorized users.

## Background

Proteins have a rich taxonomy of families and subfamilies, for which the definition and use of class labels is necessary. The adscription of a protein to a family may be uncertain, or even wrong, thus becoming an instance of what has come to be known as a label noise (LN) problem. Label noise, which is commonplace in many scientific domains [1], has a potentially negative effect on any quantitative analysis of proteins that requires the use of label information. In fact, there are few domains in which the effects of LN are so pervasive as in biomedicine and bioinformatics [2]. The problem of LN may take many forms: from the human expert subjectivity in the labelling process, which is difficult to avoid, to bounds on the available information and communication noise [3].

In medicine, for instance, the reliability of diagnostic labels is often bounded by the natural limitations of the specialists’ expertise [4], or even by the formal requirements of majority-based decision-making procedures, or consensus guidelines (for the latter see, for instance, [5]). In bioinformatics, protein subtype characterization is a task that is riddled with this problem, despite good practices in curation of genomic and proteomic databases [6].

In the specific field of G protein-coupled receptors (GPCRs), which are the target of the current study, this problem is magnified by the fact that subtyping can be performed at up to seven levels of detail [7]. GPCRs are cell membrane proteins of relevance both to biology, due to their role in transducing extracellular signals and regulating signaling pathways, and to the pharmaceutical industry for being the target for many new therapies, including pathologies related to the cardiovascular, neural, endocrine, and immune systems, as well as in cancer [8].

The current study concerns class C of these receptors, which has become an increasingly important target for new therapies, particularly in various central nervous system disorders such as Alzheimer disease, anxiety, drug addiction, epilepsy, pain, Parkinson’s disease and schizophrenia [9]. Whereas all GPCRs are characterized by sharing a common seven transmembrane helices (7TM) domain, responsible for G protein activation, most class C GPCRs include, in addition, an extracellular large domain, the Venus Flytrap (VFT) and a cystein rich domain (CRD) connecting both [10]. The VFT comprises two opposing lobes with a cleft where endogenous ligands bind. Significant synthetic efforts are currently devoted by academia and pharmaceutical companies to the design of compounds that, by binding to the 7TM domain, modulate the function of endogenous ligands allosterically. This multi-domain structural and functional complexity makes class C GPCRs an atractive target for both basic and applied (drug discovery) research. It is worth noting that, although no GPCR allosteric modulators have yet been approved for psychiatric or neurological disorders, a number of GPCR allosteric modulators including, particularly, some from class C, are under clinical development [11]. Allosteric modulators are of especial interest in comparison to orthosteric ligands due to their reduced desensitization, tolerance and side effects as well as higher selectivity among receptor subtypes and activity depending on the spatial and temporal presence of endogenous agonist [11].

Class C has been further subdivided into seven subtypes [12]: Metabotropic glutamate (mG), Calcium sensing (CS), GABA _*B*_ (GB), Vomeronasal (VN), Pheromone (Ph), Odorant (Od) and Taste (Ta) receptors. mG receptors are activated by the glutamate amino acid (AA), which is the major excitatory neurotransmitter in the brain; they comprise eight subtypes (mGlu1 to mGlu8) in turn separated into three groups: Group I (mGlu1 and mGlu5), Group II (mGlu2 and mGlu3) and Group III (mGlu4, mGlu6, mGlu7 and mGlu8). Group I mGs signal through Gq whereas Groups II and III signal through Gi/Go signaling pathways. The mG receptors are involved in major neurological disorders such as Alzheimer’s and Parkinson’s diseases, Fragile X syndrome, depression, schizophrenia, anxiety, and pain [13]. It is noteworthy that, although development programs related to the mG drugs *Pomaglumetad* (Lilly), *Mavoglurant* (Novartis) and *Basimglurant* (Roche) for the treatment of schizophrenia, Parkinson’s disease and Fragile X syndrome have recently been discontinued, some of these drugs are still expected to be beneficial for targeted patient sub-populations with neurological and psychiatric disorders [14].

The CS receptor is activated by the calcium ion and plays a key role in the regulation of extracellular calcium homeostasis. Abnormalities of the extracellular calcium sensing system lead to a disease exhibiting abnormal secretion of parathyroid hormone and hypo- or hypercalcemia. *Cinacalcet* is a marketed positive allosteric modulator of the CS receptor that has proved useful for primary or secondary hyperparathyroidism [9].

The metabotropic GB receptor is activated by GABA, a neurotransmitter which mediates most inhibitory actions in the nervous system. From a structural point of view, the GB receptor distinguishes itself from other class C GPCRs for its lack of CRD. The GB receptor is involved in chronic pain, anxiety, depression and addiction. *Baclofen* is an orthosteric agonist of the GB receptor that is commonly used as a muscle relaxant in multiple sclerosis and as analgesic. Because of their recognized pharmacological advantages, a number of positive allosteric modulators of the GB receptor are currently the goal of programs under development [9].

The investigation of protein functionality and signalling mechanisms is often based on the knowledge of crystal 3-D structures. In eukaryotic cell membrane proteins such as GPCRs, this knowledge is partial and fairly recent: The first GPCR crystal 3-D structure was fully-determined in 2000 [15] and only over the last decade, the structures of some other GPCRs, most belonging to class A, have been solved [16].

No class C full 3-D structure has yet been solved. Up until the time of writing, only two transmembrane (TM) domains and several extracellular domains of class C GPCRs have been independently determined [17, 18]. This means that, in the absence of tertiary structure information, investigations on their functionality from the primary structure (that is, from the AA sequences), in this case publicly available from several curated databases, can be particularly useful.

As mentioned in previous paragraphs, Class C GPCRs belong to different subtypes, with their corresponding labels. The occurrence of LN is unavoidable in this context because the assignment of individual sequences to one of these subtypes is itself, in most cases, a model-based process, which follows a complex many-step procedure that can only guarantee limited success [19].

GPCR subtype discrimination, as a computer-based automated classification procedure, may use aligned (through Multiple Sequence Alignment, or MSA [20]) or unaligned [21] versions of the sequences. One approach to sequence alignment-free analysis is the transformation of the primary sequences according to the physicochemical properties of their constituent AAs. Transformation methods based on the sequence composition information result on data feature vectors whose processing entails comparatively low computational costs. A review of several such methods can be found in [22].

Building from preliminary results presented in [23], we focus our investigation on the classification of data resulting from several alignment-free transformations of class C GPCR sequences, using Support Vector Machines (SVM). The sequences with the most consistent misclassification patterns are further analyzed to discover non-random LN effects, as a way to explore their possible biological explanation.

The candidate class C GPCR mislabelings detected using such approach are further validated through sequence visualization with phylogenetic trees (PT), dendrogram-like graphical representations of the evolutionary relationship between taxonomic groups which share a set of homologous sequence segments [24]. The visualization of the evolutionary relationship through PTs helped in this study to confirm the correctness of the detected persistent mislabelings.

The reported experiments using data from a curated GPCR database are meant to be the proof of concept for a systematic approach to assist the discovery of GPCR database labelling quality problems, which would in turn become the core of a label filtering decision support system [3], a useful tool for database curators in proteomics.

The remainder of the paper is structured as follows: The next section describes the analyzed class C GPCR data and the data transformation and classification methods, including the validation procedure. This is followed by a report of the experimental results and their discussion. The study wraps up with some conclusions.

## Methods

This section starts with a brief description of the data analyzed in our experiments, which is followed by an explanation of the machine learning-based classification procedure used in their analysis.

### Materials

As described in the previous section, GPCRs are cell membrane proteins with the main role of signal transmission between the intracellular and extracellular spaces. The GPCRDB is a curated, publicly accessible “molecular-class information system that collects, combines, validates and stores […] data on G protein-coupled receptors” [12].

This database divides the GPCR superfamily into several major classes, namely A (rhodopsin like), B (secretin like), C (metabotropic glutamate/pheromone), cAMP receptors, vomeronasal receptors (V1R and V2R) and Taste receptors T2R, based on the ligand types, functions and sequence similarities. Also as previously mentioned, the current study focuses on class C GPCRs.

The primary sequence data analyzed in this study were extracted from GPCRDB version 11.3.4, as of March 2011, and comprise a total of 1,510 class C GPCR sequences, belonging to the seven aforementioned subtypes (mG, CS, GB, VN, Ph, Od and Ta), including: 351 mG, 48 CS, 208 GB, 344 VN, 392 Ph, 102 Od and 65 Ta receptors. The lengths of these sequences varied from 250 to 1,995 AAs.

### Methods - alignment-free data transformations

As the AA primary sequences have a variable length, it is necessary to transform the sequence data to fixed-size vectors in order to use them with supervised classifiers. Here, we describe the different transformation methods applied to the analyzed class C GPCR dataset.

In this study, four different transformations were used, where we distinguish between those based on the *N*-gram representation built on the AA alphabet and those based on the physicochemical properties of the AAs. The use of the *N*-gram representation is common in protein characterization and has been investigated in, for instance, [25–27]. Here, we use the AAC and Digram methods, which transform the data according to the frequency of appearance of N-grams of, in turn, length one and length two in the sequence. On the other hand, we decided to use more complex transformations based on the physicochemical properties of the AAs and the sequencing information such as Auto-Cross Covariance (ACC) [28] and Physicochemical Distance-Based Transformation (PDBT [22]). Beyond computational convenience, the use of transformations based on the physico-chemical properties of the AAs is justified by the fact that, as stated in [22], “because protein structure and function are more conserved during evolutionary process, the similarity between two distantly related proteins may lie in the physicochemical properties of the AAs rather than the sequence identities”. In the following, we describe each of the transformations in some detail:

- ***N*** **-gram representations**: These transformations partially disregard sequential information to reflect only the relative frequency of appearance of AA subsequences. In the case of AAC, the frequencies of appearance of the 20 AAs (1-gram) are calculated for each sequence (i.e., a *N*×20 matrix is obtained, where *N* is the number of items in the dataset). In the case of the Digram (2-gram) method, we calculate the frequency of each of the 400 possible AA pair combinations from the AA alphabet (i.e., a *N*×400 matrix is obtained).
- **Auto cross covariance transformation**: The ACC [28, 29] is a more sophisticated transformation, capturing the correlation of the physico-chemical descriptors along the sequence. First, the physico-chemical properties are represented by means of the five *z*-scores of each AA, as described in [30]. Then, the Auto Covariance (AC) and Cross Covariance (CC) variables are computed on this first transformation. These variables measure, in turn, the correlation of the same descriptor (AC) and the correlation of two different descriptors (CC) between two residues separated by a lag along the sequence. From these, the ACC fixed length vectors can be obtained by concatenating the AC and CC terms for each lag value up to a maximum lag, *l*. This transformation generates a *N*×(*z* ^2^·*l*) matrix, where *z*=5 is the number of descriptors. In this work we use the ACC transformation for a maximal lag value of *l*=13, which was found in [31] to provide the best accuracy for the analyzed data set.
- **Physico-chemical distance-based transformation**: The PDBT transformation [22] is a complex transformation that uses a large set of physicochemical properties: 531 values representing physicochemical and biochemical properties of AAs are taken into account. Furthermore, sequence-order information is incorporated in the representation in the form of the correlation of each property between two AAs separated by a maximal lag *l*. In the current study, we use the PDBT transformation for a maximal lag of 8, which yields a *N*×4248 matrix that was previously analyzed in [32].

### Methods - SVM-based classification

SVMs have become commonplace in different problems related to the classification of proteins from their primary sequences. A non-exhaustive list of examples includes SVM-HUSTLE [33], SVM-I-sites [34], SVM-n-peptide [35], and SVM-BALSA [36]. In [22, 37], SVMs were reported to be top-performing techniques for the classification of sequences from similar transformations to those used in the current study.

These methods have their foundations on statistical learning theory and were first introduced in [38]. They map the *D*-dimensional vectors **x**_*i*_,*i*=1,…,*N*, where $\mathbf {x}_{i} \epsilon \mathbb {R}^{D}$ and *N* is the number of instances, into possibly higher-dimensional feature spaces by means of a function *ϕ*. The goal is finding a linearly-separating hyperplane that discriminates the feature vectors according to class label with a maximal margin, while minimizing the classification error *ξ*.

The most simple version is the linear SVM, where a linear hyperplane that separates the examples from two classes is assumed to exist. Such hyperplane is defined by a set of points **x** that satisfy **w**·**x**−*b*=0, where **w** is a normal vector to the hyperplane and $\frac {b}{||\mathbf {w}||}$ is the perpendicular distance from the hyperplane to the origin. In consequence, the SVM algorithm, when searching for the hyperplane with largest margin, assumes that *y*_*i*_(**x**_*i*_·**w**+*b*)−1≥0,∀*i*, where *y*_*i*_ are the class labels. The objective of the SVM algorithm is finding the separating hyperplane that satisfies this expression while minimizing ||**w**||^2^. This problem can be translated to a Lagrange formulation in which the following objective function *L*_*p*_ (primal Lagrangian) must be minimized with respect to **w**, b:

(1) $$ L_{P}\equiv \frac{1}{2}||\mathbf{w}||^{2}-\sum_{i=1}^{l}\alpha_{i}y_{i}(\mathbf{x}_{i}\cdot \mathbf{w}+b)+\sum_{i=1}^{l}\alpha_{i}   $$

This is equivalent to the maximization of the dual Lagrangian form *L*_*d*_:

(2) $$ L_{D}=\sum_{i}\alpha_{i} - \frac{1}{2}\sum_{i,j}\alpha_{i}\alpha_{j}y_{i}y_{j}\mathbf{x}_{i}\cdot \mathbf{x}_{j}   $$

subject to the restriction that **w** and b vanish (and all *α*_*i*_≥0), which leads to a closed solution.

A modification of the algorithm was introduced in [39], allowing a so-called “soft-margin” to account for mislabeled data when a linear separating hyperplane could not be found. A classification error *ξ* is admitted and a parameter *C* controlling the trade-off between those errors and margin maximization is defined (Note that, for *C*→*∞*, the model becomes equivalent to a hard-margin SVM).

The SVM can be extended to nonlinear classification [40] by applying the so-called kernel trick [41]. The use of nonlinear kernel functions allows SVMs to separate input data in higher-dimensional feature spaces in a way they would not be separable with linear classifiers in the original input space. The use of kernel functions allows to solve the problem without explicitly calculating the mapping *ϕ* (that is, without calculating data coordinates in the implicit feature space). This is possible due to the following property: *k*(**x**_*i*_,**x**_*j*_)=*ϕ*(**x**_*i*_)·*ϕ*(**x**_*j*_), which means that any dot product in the optimization procedure can be replaced by a nonlinear kernel function *k*. In this study we use the radial basis function (RBF) kernel, specified as $k(\mathbf {x}_{i},\mathbf {x}_{j})=e^{\left (-\gamma ||\mathbf {x}_{i}-\mathbf {x}_{j}||^{2}\right)}$, which is a popular nonlinear choice for SVM and has been used in the experiments reported in the following sections. With it, the model requires adjusting two parameters through grid search: the error penalty parameter *C* and the *γ* parameter of the RBF function, which regulates the “space of influence” of the model support vectors and, therefore, controls overfitting.

The discrimination of the seven subtypes of class C GPCRs requires extending the original binary (two-class) classification approach of SVMs to a multi-class one. To that end, we chose the “one-against-one” approach to build the global classification model, implemented as part of the LIBSVM library [42].

This approach performs class prediction according to the results of a voting scheme applied to the binary classifiers, i.e., according to the number of times a class is predicted in each binary classifier. Therefore, this multi-class classifier internally uses *K*(*K*−1)/2 binary classifiers for distinguishing *K* classes. A total of 21 binary classifiers were thus built for the 7 class C GPCR subtypes in our study.

#### Classification performance measures

Two different figures of merit were used to evaluate the test performance of the multi-class trained classifiers, namely the Accuracy (Accu), which is the proportion of correctly classified instances, and the Matthews Correlation Coefficient (MCC), which involves all the elements of the confusion matrix [43] and it is therefore considered a more complete figure of merit; being defined as a correlation coefficient between the observed and the predicted classification its value ranges from –1 to 1, where 1 corresponds to a perfect classification, 0 to a random classification and –1 to complete misclassification.

In our experiments, we measure the Precision, Recall and MCC at class or subtype level (i.e. at the level of the binary classifier) and measure the Accuracy and MCC at the global level (i.e., at the level of the multi-class classifier). All these figures of merit, described in Tables 1 and 2, are based on the concept of true and false predictions in binary classification with “positive” and “negative” classes. True positives (*tp*) an true negatives (*tn*) are correctly classified cases of, in turn, the positive and negative classes. Accordingly, false positives (*fp*) an false negatives (*fn*) are misclassified cases of, in turn, the negative and positive classes.

**Table 1:** Performance measures for binary classifiers

Measure	Formula	Meaning
Accuracy	$\frac {tp+tn}{tp+fn+fp+tn}$	Measure of correctness
Precision	$\frac {tp}{tp+fp}$	Measure of quality
Recall	$\frac {tp}{tp+fn} $	Measure of completeness
MCC	$\frac {tp*tn-fp*fn}{\sqrt {(tp+fp)(tp+fn)(tn+fp)(tn+fn)}}$	Correlation coefficient

**Table 2:** Performance measures for multi-class classifiers. *t* *p*_*i*_, *t* *n*_*i*_, *f* *p*_*i*_ and *f* *n*_*i*_ are, in turn, *tp*, *tn*, *fp* and *fn* for class *i* [59]. The multi-class MCC is calculated taking into account all the entries of the confusion matrix *C*_*K*×*K*_ involving all *K* classes [60]. The *ij*-th entry (*c*_*ij*_) is the number of examples of the true class *i* that have been assigned to the class *j* by the classifier

Measure	Formula	
Accuracy	$\frac {\sum _{i=1}^{K}\frac {tp_{i}+tn_{i}}{tp_{i}+fn_{i}+fp_{i}+tn_{i}}}{K}$	
MCC	$\frac {\sum _{k,l,m=1}^{K}C_{\textit {kk}}C_{\textit {ml}} - C_{\textit {lk}}C_{\textit {km}}}{\sqrt {\sum _{k=1}^{K}\left [\left (\sum _{l=1}^{K}C_{\textit {lk}}\right)\left (\sum _{f,g=1 f\not =k}^{K}C_{\textit {gf}}\right)\right ]}\sqrt {\sum _{k=1}^{K}\left [\left (\sum _{l=1}^{K}C_{\textit {kl}}\right)\left (\sum _{f,g=1 f\not =k}^{K}C_{\textit {fg}}\right)\right ]}} $	

By using a 5-fold cross-validation (CV) procedure to evaluate the multi-class trained classifier, the reported measures are the mean values of the respective metrics over the five iterations of the 5-CV.

### Methods - A systematic approach to GPCR misclassification analysis

Given a transformed dataset, our proposed systematic approach to the analysis of the classification errors consists of three steps or phases:

1. Estimation of the frequency of misclassification of each pattern (sequence) using different SVM models to select a subset of frequently misclassified patterns.
2. For each pattern in the subset selected in step 1, evaluation of the relation of votes of all the SVM classifiers between its true (label) class and its most-predicted class.
3. For each pattern in the subset selected in step 1, assessment of the decision values of the SVM binary classifiers between its true (label) class and its most-predicted class.

The aim of the first step is the detection of those patterns that, most of the times, are not classified as belonging to the class defined by their formal database label, but without considering the distribution of predicted classes in the misclassifications. Instead, the aim of the second and third steps is to confirm the consistency of the misclassifications to the most-predicted class. The difference between the two last steps resides on whether only the votes (i.e. the binary decisions of the SVM classifiers) are taken into account, or also the confidence (i.e. the decision values) of the binary SVM classifiers, when confronting just the class label against the most-predicted class, are taken into account. The union of patterns obtained as a result in steps 2 and 3 forms the final subset of frequently and consistently misclassified sequences that are shortlisted as label noise candidates.

In the following subsections, further details of each one of the three steps are provided.

#### Repeated classification with different SVM models

The first phase entails repeating the following procedure 100 times. Although this constant value could be changed, 100 is adequate both to obtain a statistically reliable result and to express the frequencies of misclassification directly as percentages (or error rates, *E* *R*_*s*_, for each sequence *s*). This type of repeated cross-validation approach has been proposed as well in [44] and applied in [45].

- First, the dataset is randomly reordered and a 5-fold cross validation (5-CV) is used, so that, for each of the five training-test partitions, the current training set is employed to construct an RBF-SVM model [42] with an optimal value for the *γ* parameter of the kernel function and with the error penalty parameter *C* varying within a small range near its previously established optimum value.
- Second, a test set classification is carried out using the trained model, registering which GPCR sequences are misclassified and generating the corresponding confusion matrix.

The use of CV in each of the 100 repetitions of this procedure ensures that each instance is classified exactly one time as a test pattern in each iteration of the outer loop. Note that *C* is slightly modified in each iteration of the inner loop.

With this, we obtain detailed results of how many times a sequence was misclassified when included in the test set and how many of these times it was assigned to specific classes. Note that all the classification results when the sequence belongs to the training set are not taken into account. In order to focus only on the most recurrent classification errors, a conservative misclassification boundary of *e*=75 *%* on the individual error rate *E* *R*_*s*_ was set (i.e., only sequences *s* misclassified in at least a 75 % of the test occasions were deemed to be strong misclassifications and selected for further analysis). This threshold *e* is merely illustrative; in a real application of the method, it should be set according to the expert analyst’s decision. A high threshold would ensure that only the most extreme misclassifications are singled out for further detailed analysis, whereas low thresholds would be more adequate in case a more global exploration is required.

#### Analysis of misclassifications according to the voting scheme

Since we are facing a multi-class (*K* classes) classification problem in which the underlying classification scheme of the SVM implementation [42] was “one-*vs*-one”, it is interesting to analyze the results of the voting scheme as applied to the *K*(*K*−1)/2 resulting classifiers, including the votes of each one, for each pattern in each test iteration. According to LIBSVM, the subtype with the highest number of votes in each case becomes the predicted class of the test pattern.

For each frequently misclassified sequence *s*, selected in the first phase, we focus the analysis on the relation between the total number of votes *V* *T*_*s*_ obtained by the true (label) class in the 100 iterations and those obtained by the most frequently predicted class for that sequence, *V* *P*_*s*_. This is, we define the voting ratio

(3) $$ R_{s} = \frac{VT_{s}}{VP_{s}}  $$

and, given some threshold *θ*_*R*_, we consider that *R*_*s*_≤*θ*_*R*_ indicates a consistent (also deemed as *large*) classification error, while *R*_*s*_>*θ*_*R*_ denotes a more doubtful (or *small*) misclassification. We fixed a threshold *θ*_*R*_=0.5 to obtain our results discussed later.

#### Analysis of misclassifications according to the decision values

In the third and last phase of our proposed approach, we go deeper into the analysis of misclassifications by taking into account the confidence (decision values) of the 100 binary SVM classifiers involving only the label class and the most frequently predicted class, when classifying a sequence *s* as test pattern. For each frequently misclassified sequence *s* selected in the first phase, we define a *cumulative decision value*, *C* *D* *V*_*s*_, as follows:

(4) $$ CDV_{s} = \sum_{k=1}^{100} DV_{s}(i,j,k)  $$

where *D* *V*_*s*_(*i*,*j*,*k*) is the decision value given by the binary SVM classifier confronting the class with label *i* to which *s* formally belongs and the most-frequently predicted class for sequence *s*, with label *j*, in the *k* ^*th*^ test iteration. GPCR subtype labels were numbered 1 to 7 in the order they are presented in the data description section. For subtypes *i*,*j*, a large positive *C* *D* *V*_*s*_ value if *i*>*j* and a large negative one if *i*<*j* both indicate clear misclassifications. Hence, the magnitude of the error is deemed *large* or *small* depending on whether the *C* *D* *V*_*s*_ exceeds a certain threshold *θ*_*CDV*_ in absolute value or not. A threshold *θ*_*CDV*_=60 was chosen for the experiments.

Note that the information conveyed by *C* *D* *V*_*s*_ complements that of *R*_*s*_. For instance, a misclassified sequence with high *R*_*s*_ would suggest that the voting process discards all subtypes but the true and the predicted ones, that is, a very narrow transfer of subtype assignment. If this is accompanied by a large *C* *D* *V*_*s*_ in absolute value, the predicted subtype, even if wrong assuming that the identifying label of the sequence is trusted, is strongly preferred by the SVM classifiers.

### Methods - External validation of SVM-based classification

#### Mislabeling validation with phylogenetic trees

For proteins, a PT is a dendrogram-like graphical representation of the evolutionary relationship between taxonomic groups which share a set of homologous sequence segments. This evolutionary relationship is a form of hierarchically structured similarity-based grouping process.

In this study, PTs were used to visualize the analyzed class C GPCR sequences and thus provide an alternative way to externally validate the misclassification results reported in the previous sections. There are two sound reasons why we use PTs for this task: first, because they have *de facto* become standard tools in bioinformatics [24] and, particularly, in protein homology detection, so that protein database curators are more likely to trust them. Second, because the protein sequence alignment that underlies the tree construction has no direct link with the sequence transformations from which the SVM classifiers are built, therefore guaranteeing the independence of the results.

Our software tool of choice, Treevolution^1^ [46], was developed in Java and integrates the Processing^2^ package. This tool supports visual and exploratory analysis of PTs in either Newick or PhyloXML formats as radial dendrograms, with high-level user-controlled data interaction at the user request and offers several methods very useful for large PT: sector distortion, tree rotation, pruning, labeling, tracking of ancestors and descendants and text search, among others.

The color-guided highlighting of protein families helps the user to focus on sequence groupings of interest and give an overall idea of groups with the same ancestor within the tree. The PT is created from a MSA obtained with Clustal Omega [47]. This application, in which sequences data are introduced in FASTA format, performs distance-based MSA [48].

## Results

This section starts with a brief summary of some preliminary research that inspired the current study. This is followed by a detailed analysis of misclassifications according to a voting scheme and classifier decision values. This analysis is validated using a standard LN detection filter and sequence visualization through PTs.

### Results from previous research

The experiments reported in this section extend some basic preliminary results reported in [23]. In previous research [49], we investigated the supervised classification of the data set described in the previous section using different classifiers, namely decision trees (DT), naïve Bayes (NB) and SVM, for different alignment-free transformations of the sequences, including AA composition (AAC), the Mean Transformation [50] and Auto-Cross Covariance (ACC) [28]. In this previous study, focus was placed on the accuracy of the classifiers’ performance and the experimental results showed that SVM clearly outperformed the rest of classifiers independently of the transformation applied to the data set. This led to the conclusion that a nonlinear classifier with the ability to find a linear separation of instances in a higher-dimensional feature space, such as SVM, was the adequate choice for the data set under analysis in the task of subtype discrimination. The second conclusion from this previous study was that, at subtype level, classification accuracies showed only small variations depending on data transformations. Even a superficial analysis of the confusion matrices showed recurrent patterns of subtype misclassification, which hinted at LN as their cause. Such observations provided support for a more detailed analysis of sequence misclassification.

### Repeated classification with different SVM models using different transformations of the dataset

**Fig. 1:**
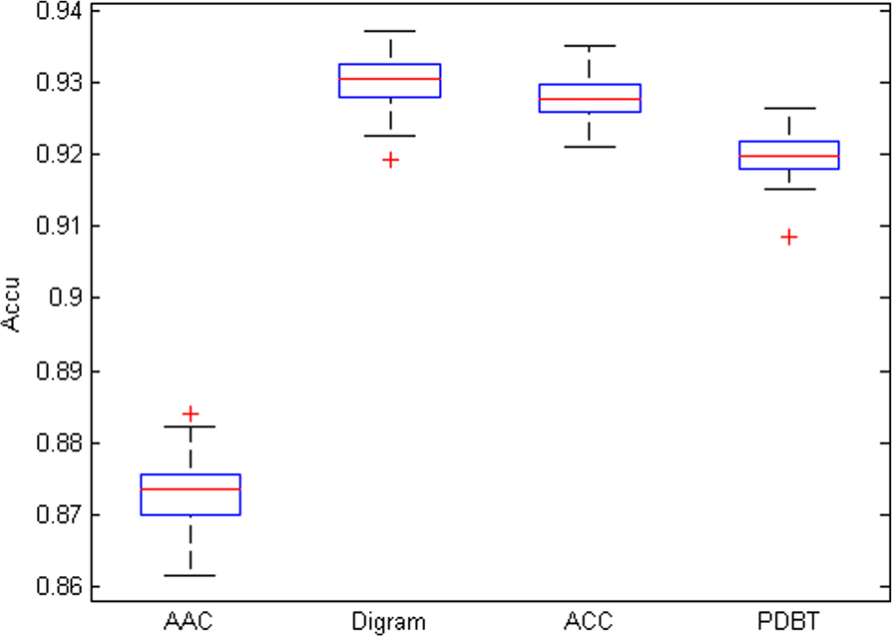
Boxplot representation of the Accu of the AAC, Digram, ACC and PDBT dataset

**Fig. 2:**
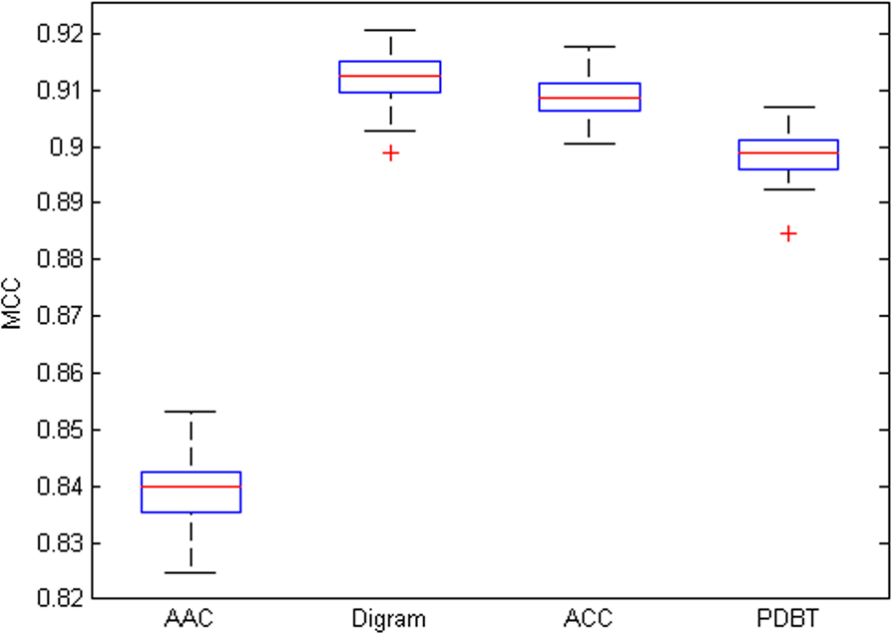
Boxplot representation of the MCC of the AAC, Digram, ACC and PDBT dataset

These previous results led us to decide on the convenience of using a more diverse set of data transformation techniques. Tables 3 and 4 summarizes the best subtype classification results obtained with SVM for the four different transformed data sets, measured by average accuracy (overall correct classification rate) and MCC. These results are complemented by the box-plot representation of the distributions of the accuracy and MCC values, for each of the transformed data sets, over the 100 outer iterations of the classification procedure, shown in Figs. 1 and 2. For all transformations, a low variability of the results is observed, suggesting consistent estimates that make the average figures of Table 3 quite reliable. Out of these, the best classification results were found for the Digram and ACC transformed data sets, although the relative differences of accuracy and MCC make PDBT also a reasonable choice.

**Table 3:** SVM classifier results: Global results for the four data transformations; accuracy (Accu), Matthews Correlation Coefficient (MCC)

Data	Accu	MCC
AAC	0.88	0.84
Digram	**0.93**	**0.91**
ACC	**0.93**	**0.91**
PDBT	0.92	0.90

Best results highlighted in bold

**Table 4:** SVM classifier results: Class C GPCR results per subtype for the ACC data set only, including MCC, Precision (Prec) and Recall (Rec)

Class	MCC	Prec	Rec
mG	0.95	0.95	0.99
CS	0.93	1.00	0.88
GB	0.98	0.99	0.99
VN	0.89	0.91	0.92
Ph	0.86	0.89	0.90
Od	0.79	0.89	0.74
Ta	0.99	1.00	0.98

A detailed analysis of the results per-subtype revealed relatively minor differences between those obtained with each of the four transformed data sets. This observation suggests that the main causes of misclassification might lie beyond the differences between data transformations and that a more systematic analysis of the classification errors is required.

Table 5 shows a few illustrative misclassification statistics for the ACC transformed data set. For instance, sequence *♯*6, which belongs to subtype *VN* according to its database label, was misclassified 100 out of 100 times: 96 of them was assigned to *Ph* and 4 to *Od* (See Table 6 for the mapping between the number *♯* and the protein database *Id*).

**Table 5:** Illustrative example of misclassification statistics for the ACC data set. For some sequences *s* identified by number *♯*_*s*_, the error rate (*E* *R*_*s*_), the true class (*T* *C*_*s*_), and how many times this sequence was misclassified as belonging to each of the other subtypes (from mG to Ta), are displayed. The three last columns list the sum of the votes for the true class (*V* *T*_*s*_), for the most frequently predicted class (*V* *P*_*s*_), and the ratio (*R*_*s*_) of one to the other

*♯*_*s*_	*E* *R*_*s*_	*T* *C*_*s*_	mG	CS	GB	VN	Ph	Od	Ta	*V* *T*_*s*_	*V* *P*_*s*_	*R*_*s*_
2	100	CS	100	0	0	0	0	0	0	91	600	0.15
6	100	VN	0	0	0	0	96	4	0	404	596	0.67
7	100	VN	100	0	0	0	0	0	0	300	600	0.5

**Table 6:** Sequences with large classification errors: For each sequence *s* numbered *♯*_*s*_, the GPCRDB Identifier (*I* *d*_*s*_), the true class (*T* *C*_*s*_), the predicted class (*P* *C*_*s*_), the *voting ratio* (*R*_*s*_) and the *cumulative decision value* (*C* *D* *V*_*s*_) are displayed

*♯*_*s*_	*I* *d*_*s*_	*T* *C*_*s*_	*P* *C*_*s*_	*R*_*s*_	*C* *D* *V*_*s*_
1	q5i5c3_9tele	mG	Od	0.75	**–95**
2	XP_002123664	CS	mG	**0.15**	50
3	q8c0m6_mouse	CS	Ph	**0.15**	–46
4	XP_002740613	CS	mG	**0**	**–66**
5	XP_002936197	VN	Ph	0.83	**–96**
6	XP_002940476	VN	Ph	0.67	**–95**
7	XP_002941777	VN	mG	**0.5**	45
8	B0UYJ3_DANRE	Ph	mG	0.79	**109**
9	XP_001518611	Od	mG	**0.31**	46
10	XP_002940324	Od	VN	0.49	**70**
11	GPC6A_DANRE	Od	Ph	**0.5**	**74**

Extreme *R*_*s*_ and *C* *D* *V*_*s*_ values highlighted in bold

This misclassification analysis was repeated for each of the transformed data sets. The AAC, Digram, ACC and PDBT sets yielded, in turn, 143, 88, 85 and 100 strong misclassifications. A detailed analysis of these frequently misclassified sequences revealed that they are nearly identical for ACC and Digram. There are some differences with the PBDT misclassifications that might be the result of the very different type of transformation. Importantly, 52 frequently misclassified sequences were common to all four data sets and there was strong agreement on the most-often predicted subtypes. These sequences are listed in Additional file 1.

### Analysis of misclassifications according to the voting scheme

Interestingly, these results suggest the existence of subtypes with recurrently wrong class assignments. So, we applied the second step of our systematic approach based on the voting scheme, as described earlier, to confirm consistent misclassifications. To illustrate the results obtained in this step, we show the voting scheme results for the selected instances of Table 5. Sequence *♯*6, for instance, is a *VN* consistently misclassified as *Ph*. The magnitude of the error is small, though, as the *voting ratio* (*R*_*s*_) of true class to predicted class is relatively high (0.67>0.5). Sequence *♯*2 is a *CS*, consistently misclassified as *mG*. The magnitude of the error is large, as the *R*_*s*_ is quite low (0.15≤0.5).

Only 7 of the 85 frequently misclassified ACC-transformed sequences yielded large errors (See Table 6). Similarly, for AAC, Digram and PDBT sets, the majority of sequences have small errors.

### Analysis of misclassifications according to the decision values

Clear differences in the magnitude of the recurrent classification errors were found. Pursuing further insight, we applied the third step of our approach based on the *cumulative decision value* (*C* *D* *V*_*s*_) specifically for the binary classifier that involves the true class and the predicted class.

As previously mentioned, the magnitude of the error was deemed *large* or *small* depending on whether the CDV exceeded the threshold of 60 in absolute value or not. A total of 21 out of the 85 frequently misclassified instances of the ACC-transformed data set have a *large* error according to this criterion, whereof 4 yield a *very large* one (|*C* *D* *V*_*s*_|≥95: see Table 6).

### Summary of the analysis of misclassifications

The proposed subtype classification approach revealed the existence of a number of instances that, independently of the sequence transformation method, induce classification errors that could be deemed either large or small. The information provided by *R*_*s*_ and *C* *D* *V*_*s*_ should be understood as complementary, given that not fully coincident instances are singled out in each approach.

Importantly, this analysis showed that the misclassifications of a sizeable proportion of sequences have a small magnitude, so that they could be ignored unless a thorough revision of the database labels is required. A small number of instances, though, showed consistent and large classification errors and they should be the focus of interest from the database curation viewpoint. In Table 6, we list GPCRs with either very large absolute value of *C* *D* *V*_*s*_ (4 items) or small *R*_*s*_ (7 items) using the ACC transformed dataset.

### Mislabeling validation

#### Validation through PT-based visualization of class C GPCRs

**Fig. 3:**
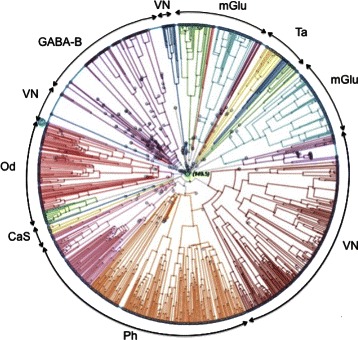
Radial PT plot showing the main areas of distribution of the seven class C GPCR subtypes. Treevolution radial PT in which the main sections occupied by each of the seven class C GPCR subtypes are explicitly represented by archs or groups of archs in the periphery of the tree. Note that branch colors are automatically generated during PT construction and do not correspond to class C subtypes

Figure 3 displays the *Treevolution* radial PT plot of the complete set of 1,510 GPCRs of class C, additionally showing the approximate distribution of its main seven subtypes. In this representation, each outer branch corresponds to one GPCR sequence. Tree colors are used to represent families of descendant nodes. Note though that these colors do not correspond to subtype labels. We observe that some families correspond to not one but several evolutionary branches. For example, the two different colors assigned to Pheromone provide quantitative evidence of the existence of at least two subtypes within the family. The representation of the evolutionary relationship in the PT plot shows that there exist some clearly separated families (GB, CS and Od), while others are more closely related to each other, such as mG and VN.

In the following, we report the PT plots for the four class C subtypes that were predicted for the mislabeling candidates listed in Table 6. In them, these potential mislabelings (those with largest errors according to the proposed approach) are highlighted (See the individual sequences listed in Table 6).

**Fig. 4:**
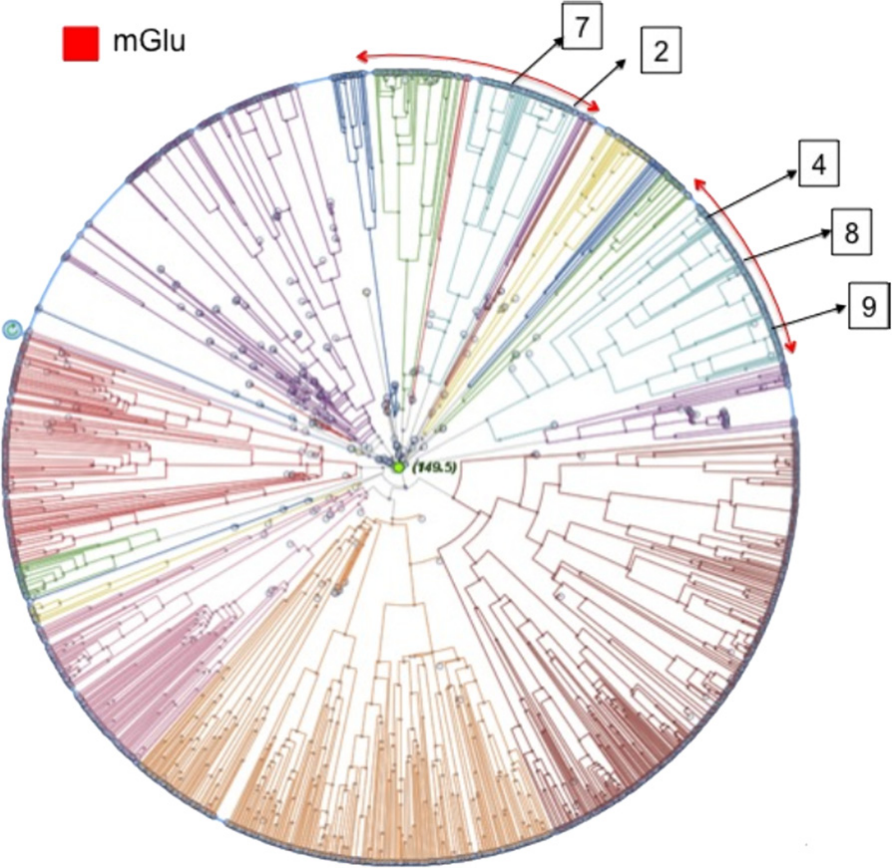
Mislabelings predicted to be mG. Five sequences with large classification errors were mislabeled as mG. Sequence *♯*7 was labeled as *VN* in GPCRDB; *♯*2 and *♯*4 were labeled as *CS*; *♯*8 was labeled as *Ph*; and *♯*9 was labeled as *Od*

Figure 4 shows the selection of sequences with largest errors that were predicted to be *mG*. The *mG* subtype has two main evolutionarily-related subgroups, which are shown schematically in the PT plot. In our analysis, we found 5 sequences with large classification error. In this PT, they are highlighted in their locations. We see that sequences *♯*7 (labeled as *VN* in GPCRDB) and *♯*2 (labeled as *C* *S*) both fall into the first area of *mG*. The instances *♯*4 (labeled as *CS*), *♯*8 (labeled as *Ph*) and *♯*9 (labeled as *Od*) fall into the second area of *mG*.

**Fig. 5:**
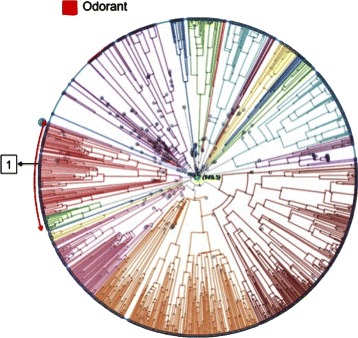
Mislabelings predicted to be Od. One sequence (*♯*1, labeled as *mG* in GPCRDB) with large classification error was mislabeled as *Od*

Figure 5 shows the single sequence with large error predicted as *Od* by the proposed mislabelling filtering approach. The Odorant subtype corresponds to a single area in the PT plot, and sequence *♯*1 (labeled as *mG*) falls clearly into this area.

**Fig. 6:**
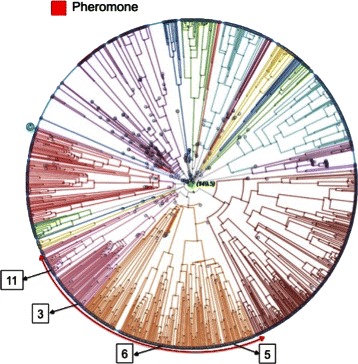
Mislabelings predicted to be Ph. Four sequences with large classification errors were mislabeled as Ph. Sequence *♯*3 was labeled as *CS* in GPCRDB; *♯*11 was labeled as *Od*; and *♯*5 and *♯*6 were labeled as *VN*

Figure 6 shows the sequences found to be *Ph* by the proposed approach. The *Ph* subtype has two main evolutionarily-related subgroups, which are shown schematically in the PT plot. Sequences *♯*11 (labeled as *Od*) and *♯*3 (labeled as *CS*) fall into the first subgroup, whereas sequences *♯*5 and *♯*6 (labeled as *VN*) fall into a separate evolutionary branch.

**Fig. 7:**
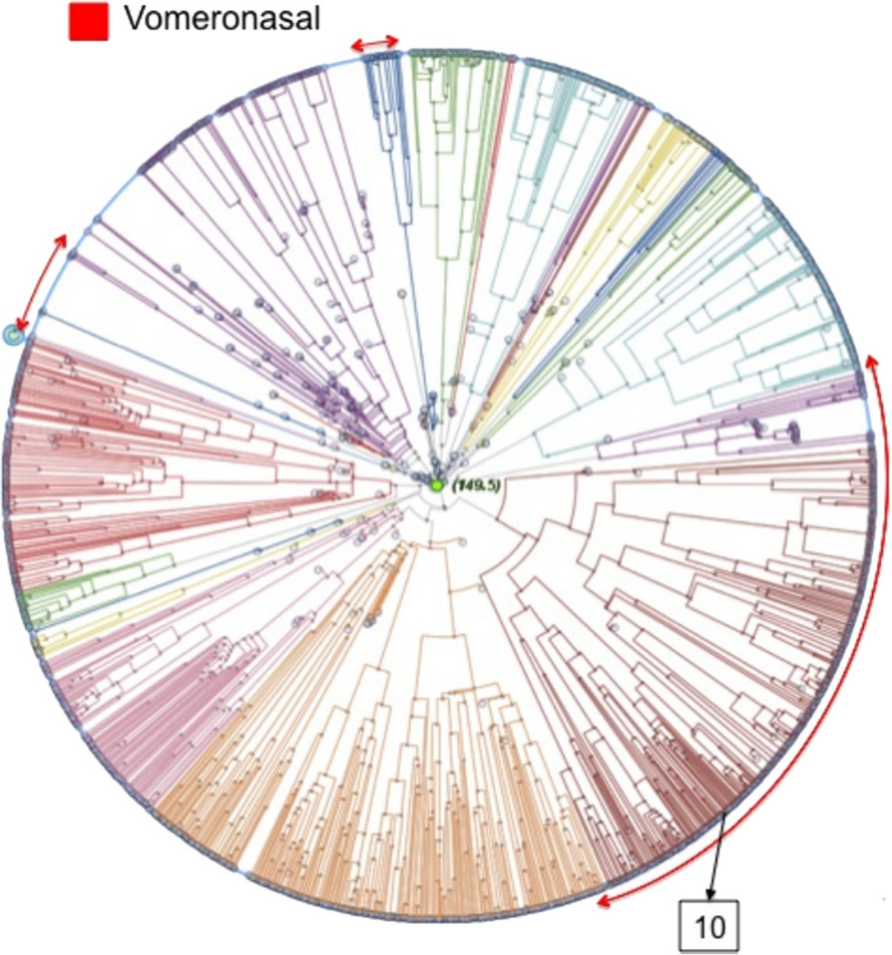
Mislabelings predicted to be Vn. One sequence (*♯*10, labeled as *Od* in GPCRDB) with large classification error was mislabeled as *Vn*

Figure 7 shows the sequence found to be *VN* by the proposed approach. The *VN* subtype corresponds to three evolutionary areas in the PT plot. Sequence *♯*10 (labeled as *Od*) falls into one of these areas.

#### Comparison with an ensemble-based noise detection approach

As mentioned in earlier sections, in previous studies we carried out classification experiments on the AAC and ACC datasets [49] using different supervised classifiers, including NB, DT and SVM. From these, we concluded that SVM classifiers significantly outperformed NB and DT for both the AAC and ACC data sets. In this section, we return to these less accurate classifiers, which are more robust to LN as they apparently carry out a more generic classification of the investigated data set, and should be less prone to data overfitting, a possible risk associated to the more accurate SVM classification models [51].

We describe here the results of the application of an ensemble-based noise detection approach including less accurate classifiers for the analysis of the class C GPCR data set. This method just detects label noise candidates by counting the misclassifications of an instance for the different classifiers in the ensemble. In some way, this is similar to the first step of our proposed approach, with the difference that in our case the ensemble is composed only by SVM classifiers. The second and third steps of the proposed approach, though, allow a more fine-grained analysis of the misclassifications by taking into account the results of the classifiers involving just the sequence class label and the most-frequently predicted class for that sequence. In fact, we do not aim to a straightforward comparison between methods, but to use the ensemble method as a way to test the coincidence on the subset of mislabeled sequences detected by both.

Ensemble-based noise detection methods have their origin in ensemble learning [52], where a set of prediction models are constructed using different algorithms and their output is combined to generate a single prediction. A noise detection ensemble classifier filter [53] consists of a set of diverse base classifiers. Their classification errors are combined to detect mislabeled instances using either a consensus vote filter (all classifiers detect a classification error), or a majority vote filter (the majority of classifiers detect an error).

In this experiment, we use an ensemble classifier built using NB, Random Forest (RF), SVM and Multi-Layer Perceptron (MLP) classifiers to analize the ACC-transformed data set. The results of the base classifiers are evaluated using a noise rank filter [54], which provides information about the ranking of detected candidates to misclassification. The rank filter estimates the following weights (in brackets) for each classifier in the calculation of the rank: MLP (3), SVM (2), NB (1) and RF (1), and assigns a ranking to the sequences according to the number of classifiers that failed to evaluate them correctly. It then reports in how many classifiers the prediction failed. In our analyses, we focused on those sequences that were evaluated incorrectly by either all classifiers (a total of 117), or by at least three of them (a further 34). We then checked which of these 151 sequences were also detected as frequently misclassified by our proposed SVM approach. A total of 141 instances were found. Both methods coincided in the detection of 109 sequences as possible mislabelings (a 77 % coincidence). All sequences with large classification error listed in Table 6 were also detected by the noise rank algorithm.

This result provides further support to the claim of effectiveness of the proposed SVM-based approach in its task as LN detector.

## Discussion

The systematic approach proposed for the analysis of the SVM misclassifications has revealed the existence of a number of sequences that, independently of the transformation method, are prone to classification errors that could be deemed large or small (according to criteria that, ultimately, should be set by proteomics experts). The information provided by the voting ratio *R* and the absolute value of the CDV should be understood as complementary, given that not fully coincident sequences are singled out by each approach; that is, some sequences might show very low values of R but not very high values of CDV, or very high values of CDV but not too low values of R.

Importantly, this analysis has shown that the misclassifications of a sizeable proportion of sequences have a small magnitude. All these sequences might well be considered as mild cases of LN and should eventually be redirected to a human expert for further analysis. Small errors also suggest underlying similarities between the GPCR subtypes whose characteristics may be unknown and worth investigating. A small number of instances, though, show consistent and large classification errors. They merit detailed study because they might be affected by a more radical type of LN, or even by straight mislabelling. These are the sequences listed in Table 6, which are now individually discussed.

Sequences *X* *P*_002123664,*X* *P*_002740613,*X* *P*_0029361 97,*X* *P*_002940476 and *X* *P*_002940324 are all recurrently misclassified. *X* *P*_002740613, in particular, yields a 100 % error, *R*=0 and large CDV. Their labels should require further expert assessment, given that they were derived by an automated computational analysis from an annotated genomic sequence by means of a gene prediction mode from the RefSeq^3^ databank. Another couple of interesting cases are *q*8*c*0*m*6_*m* *o* *u* *s* *e* and *B*0*U* *Y* *J*3_*D* *A* *N* *R* *E*. According to the information referenced at UniProt^4^, these GPCRs are unreviewed and should be considered only as preliminary data. The former, according to GPCRDB, is a *CS* that our system confidently (*R*=0.15) classifies as *Ph*. The European Nucleotide Archive^5^ lists it as similar to the putative *Ph* receptor V2R2. The latter, according to GPCRDB, is a *Ph*, while our system predicts it to be an *mG* with a very large CDV (109). Agreeing with our prediction, the Ensembl Genome Browser^6^ considers it to be an *mG* of subtype 6*a*.

Sequence *G* *P* *C*6*A*_*D* *A* *N* *R* *E* is labeled as *Od*, but the low number of votes of this class and the large CDV suggest its classification to *Ph*. Although this sequence is considered as olfactory receptor^7^, we suggest to investigate the possibility of its labelling as *Ph*.

As stated in the previous section, it is important to provide further validation for the clearest of the misclassifications found with the proposed method (as summarized in Table 6) using PTs. The importance of this validation resides in the fact that the PT dendrograms are not built from the same data transformations we used. Therefore, agreement between the subtype assignment of the PT and the label predicted by our method should be an almost definitive confirmation of the existence of label noise, whereas, contrarily, lack of agreement might be an indication that the misclassification is caused by the type of sequence transformation itself, or by the fact that the subtypes defined by the existing and predicted labels overlap.

The comparison of the most extreme misclassifications discovered with the proposed method with the visual results provided by the PTs (See Figs. 4, 5, 6 and 7) is striking, as it provides consistent evidence of the reliability of the former. Figs. 4, 5, 6 and 7 show that the detected extreme mislabellings fall exactly into the evolutionary branch belonging to the class predicted by the proposed approach. This reliability is a guarantee that the method is viable as a tool for database curators in proteomics.

## Conclusions

Label noise is a potentially important problem in the process of automated class C GPCR subtype classification from the alignment-free transformed versions of protein primary sequences. This is because the labels of these sequences are obtained indirectly through complex, many-step similarity modelling processes.

In this paper, we have proposed a systematic procedure, based on SVM classification, to single out and characterize GPCR sequences with consistent misclassification behaviour. This approach, where the detection of possible mislabeled data is based on the analysis of the frequency of misclassification of an instance and a quantitative assessment of the magnitude of the classification error, has been applied to different sequence data transformations and shown to be a viable alternative for the definition of a prediction-based system addressing the problem of label noise.

For a database like the one analyzed in the current study, the type of label noise is well-defined within the general taxonomy of the problem [1]: it should not be mistaken by a problem of outlier or anomaly detection and can be considered as the natural result of human expert involvement and model-based (semi)automated labeling [55]. As such, it falls within the *noisy not at random* type of models, because sequences are more likely to be mislabeled when they are similar to sequences of other subtype and because labels are likely to be less certain in regions of low data density. Mislabeling thus depends both on the data features and on the true labels. Three general (and partially overlapping) approaches are available to tackle this problem: the use of classification algorithms that are robust to label noise; the use of *filter* methods that detect *noisy* cases; and the use of algorithms for explicit label noise modeling. A large palette of methods has been proposed for each of these and their review is beyond the scope of this study. The reader is referred to [1] for an up-to-date survey.

Here, our choice was a variant of a filtering approach, because, as acknowledged in [1], “some of the label noise-tolerant variants of SVMs could also be observed as filtering”. The proposed method can therefore be considered as model predictions-based filtering [56], extending the basic concept of voting filtering [53, 57] and attempting to improve model robustness by decomposing a multi-class problem into multiple binary classification problems [58]. The reported experimental results are a proof of concept for the viability of such procedure as part of a decision support system that, combined with expert knowledge in the field, should be able to assist the discovery of GPCR database labelling quality problems. These results have been further validated using PTs, a standard tool in bioinformatics.

In future research, we aim to explore alternative approaches to the label noise problem and plan to extend this work to implement the proposed method as a publicly-available software tool with a user-friendly GUI for bioinformatics scientists.

## Endnotes

^1^ http://vis.usal.es/treevolution

^2^ http://processing.org

^3^ http://www.ncbi.nlm.nih.gov/refseq/

^4^ http://www.uniprot.org/uniprot/B0UYJ3, http://www.uniprot.org/uniprot/Q8C0M6

^5^ http://www.ebi.ac.uk/ena/data/view/BAC26854

^6^ http://www.ensembl.org

^7^ http://www.uniprot.org/uniprot/Q5U9X3

## Additional file

Additional file 1
**This is a** ***csv*** **format file containing the table of 52 frequently misclassified sequences that were common to all four data transformations, as described in the** ***Results*** **section of the main text.** It includes the following columns: GPCRDB identifier, GPCRDB *true* class, and predicted class for, in turn, the AAC, Digram, ACC and PDBT transformations. A strong agreement on the most-often predicted class C GPCR subtypes can be observed. (XLS 22 Kb)

## Abbreviations

AA: Amino acid
AC: Auto covariance
ACC: Auto cross covariance
Accu: Accuracy
CC: Cross covariance
CDV: Cumulative decision value
CRD: Cystein rich domain
CS: Calcium sensing
CV: Cross validation
DT: Decision tree
DV: Decision value
ER: Error rate
fp: False positive
fn: False negative
GB: GABA _*B*_
GPCR: G Protein-coupled receptor
Id: Identifier
L _*p*_: Primal lagrangian
L _*d*_: Dual lagrangian
LN: Label noise
MCC: Matthews correlation coefficient
mG: Metabotropic glutamate
MLP: Multi-layer perceptron
MSA: Multiple sequence alignment
NB: Naïve bayes
Od: Odorant
PC: Predicted class
PDBT: Physicochemical distance based
Ph: Pheromone
Prec: Precision
PT: Phylogenetic trees
R: Voting ratio
RBF: Radial basis function
Rec: Recall
RF: Random forest
SVM: Support vector machine
Ta: Taste
TC: True class
TM: Transmembrane
tn: True negatives
tp: True positives
VFT: Venus flytrap
VN: Vomeronasal
VT: Total number of votes
VP: Number of votes of the predicted class
5-CV: 5-fold cross validation
7TM: Seven transmembrane helices
